# Enucleation of the Prostate

**Published:** 1901-08-10

**Authors:** 


### ENUCLEATION OF THE PROSTATE.

In a clinical lecture recently delivered at the Polyclinic, Dr. P. J. Freyer described and advocated an operation which he has lately practised for the complete enucleation of the prostate gland. Of the various operative methods which have hitherto been used for the relief of prostatic obstruction he gives the preference to that introduced into England by McGill, but even after this operation a bladder which has once lost its contractility often fails to regain its expulsive power. The fact is that the outgrowth into the bladder which it is the object of McGill's operation to remove is not the sole cause of the obstruction. This is mainly due to the pressure of the enlarged lateral lobes on the urethra. With the object, then, of overcoming this difficulty Dr. Freyer has in four recent cases enucleated the whole of the prostate gland, or rather, glands, with complete success. He points out that the prostate is really composed of twin organs which are separate and distinct during the first four months of fœtal life. After that period they approach each other and their inner surfaces become agglutinated together, except along the course of the urethra, which they envelop in their embrace. But although welded together in this way these two organs remain, so far as their secreting substance, ducts, and functions are concerned, as distinct as the testes. After opening the bladder suprapubically and incising the mucous membrane over the tumour caused by the enlarged prostate, it is possible to enucleate the mass lobe by lobe and through separate incisions if necessary, and the gland being thus enucleated in its capsule, whether it comes away as a whole or in separate lobes, is stripped off the urethra, which with its enveloping tissues is left intact. In enucleating the prostate out of its sheath the fibrous bands that pass between the sheath and the true capsule are torn through, but the prostatic plexus of veins and the larger arteries are left behind, only the smaller vessels passing to and from the prostatic substance through the capsule being severed. Thus the hæmorrhage is not great, far less indeed than that which often takes place in partial prostatectomy. Certainly this operation marks a great advance, for none of the older methods gave any security against a recurrence of the obstruction from the continued progress of the disease in the remaining portions of the prostate. Moreover, in regard to the restoration of the power of expulsion, this operation stands on a very different plane from those incomplete methods which have hitherto been our sole resource when catheterism has failed.

